# The Royal Medical and Chirurgical Society

**Published:** 1835-01-01

**Authors:** 


### INTELLIGENCE.

The following document, by which a charter is conferred on the greatest of our medical societies, seems worthy of being recorded in our pages.

### CHARTER.

William the Fourth, by the Grace of God of the United Kingdom of Great Britain and Ireland King, Defender of the Faith,—To all to whom these presents shall come greeting.

Whereas John Elliotson, doctor of physic, Sir Astley Paston Cooper, baronet, and John Yelloly, doctor of physic, have, by their petition, humbly represented unto us that a society was formed in the year 1805, by a considerable number of physicians and surgeons of eminence in London, for the cultivation and promotion of physic and surgery, and of the branches of science connected with them, of which the two last-named of the petitioners were original members; and that the said society has expended considerable sums of money in the purchase and collection of a large and valuable library, and has published eighteen volumes of Transactions, which have had a very extensive circulation. And whereas they, the said petitioners, have humbly besought us that we should give to them, and to the other persons who have already become members of the said society, or who may at any time hereafter become members of our Royal Charter of Incorporation, for imparting greater stability and effect to the designs of the said society. Now know ye that we, being desirous of encouraging a design so laudable, have, of our special grace, certain knowledge, and mere motion, willed, granted, and ordained, and do by these presents, for us, our heirs and successors, will, grant, and ordain that the said John Elliotson, Sir Astley Paston Cooper, and John Yelloly, and such others of our loving subjects as are now members of the said society, or who shall at any time hereafter become members thereof, according to such bye-laws as shall hereafter be framed or enacted, shall by virtue of these presents be called fellows of the said society, aud shall be one body politic and corporate, by the name of "The Royal Medical and Chirurgical Society of London;" of which society we do hereby declare ourselves and successors, if they shall think fit, the patron, by which name they shall have perpetual succession, and a common seal, with full power to alter, vary, break, and renew the same at their discretion, and by the same name to sue and be sued, to implead and be impleaded, to answer and be answered unto, in every court of us, our heirs, and successors, and be for ever able and capable in the law to purchase, receive, hold, possess, and enjoy, to them and their successors, any goods and chattels whatsoever, and also be able and capable in the law (notwithstanding the statutes of mortmain,) to take, purchase, hold, and enjoy, to them and their successors, any lands, tenements, or hereditaments whatsoever, the yearly value of which shall not exceed in the whole the sum of two thousand pounds, computing the same respectively at the rackrent which might have been had or gotten for the same respectively at the time of the purchase or acquisition thereof; and shall have full power and authority to sell, alien, charge, or otherwise dispose of any real or personal property so to be by them acquired as aforesaid, and to act and do in all things relating to the said corporation in as ample manner and form as any other our liege subjects, being persons able and capable in the law, or any other body politic and corporate in our said United Kingdom of Great Britain may or can act or do.

And we do further declare and grant that, for the better government of the said society, and for the better management of the concerns thereof, there shall be, from the date of these presents thenceforth and for ever, a president of the said society, who with twenty fellows, to be elected in manner hereinafter mentioned, shall form the council. And we do hereby appoint the said John Elliotson the first president of the said society, and the said Sir Astley Paston Cooper and John Yelloly the first members of the council, to continue in office till the 1st day of March next. And we further direct that, within four months from the date of these letters patent, a general meeting of the fellows of the said society shall be held, who shall be authorised by method of ballot to elect eighteen fit and proper persons as officers or other members of the council, to complete the number of twenty-one, of whom, including the president, we have willed that the council shall be composed, and that such additional persons shall likewise continue in office till the 1st day of March next, and till other fit and proper persons be chosen in their room.

And our further will and pleasure is, that the fellows of the said society shall and may on the 1st day of March, 1835, and also shall and may on the 1st day of March in every succeeding year, or as near the same as conveniently may be, assemble together at the then last or other usual place of meeting of the said society, and proceed by method of ballot to nominate and appoint a president of the said society, and such officers and other members of the council as may with the president form the number of twenty-one, of whom we have willed that the council shall consist; and also may, in case of the death, resignation, or removal of the president, or any officer or other member of the council, within the space of three months next after such death, resignation, or removal, elect some other person, being a fellow of the said society, to supply the place of such president, or officer or other member of the council. And our further will and pleasure is, that no fellow who has filled the office of president for two successive years shall be again eligible to the same situation until the expiration of one year from the termination of his office, and that not more than two thirds of the fellows who have formed the council of the preceding year shall be re-elected members of the council at such annual meeting. And we do further grant and declare that the fellows of the said society, or any ten or more of them, shall and may have power from time to time, at the meetings of the said society, to be held at the usual place of meeting of the said society, or at such place as shall have in that behalf been appointed, by and with the consent of not less than four fifths of the fellows present, to elect such persons to be fellows of the said society, and all fellows to remove from the said society as they shall think fit; and that the council hereby directed to be appointed, and the council of the said society for the time being, or any three or more of them, all the members thereof having been first duly summoned to attend the meetings thereof, shall and may have power, according to the best of their judgment and discretion, to make and establish such bye-laws as they shall deem proper and necessary for regulating the affairs of the said society, and also the number and description of its officers, and also the times, place, and manner of electing and removing the fellows of the said society, and all such subordinate servants, officers, and attendants as shall be deemed necessary or useful for the said society, and also for filling up from time to time any vacancies which may happen by death, resignation, removal, or otherwise, in any of the officers or appointments constituted or established for the execution of the business and concerns of the said society, and for regulating and ascertaining the qualifications of persons to become fellows of the said society respectively, and also the sum and sums of money to be paid by them respectively or any of them, whether upon admission or otherwise, towards carrying on the purposes of the said society, and also the number, qualifications, and privileges of such persons as they may from time to time deem it proper to admit as honorary fellows; and such bye-laws from time to time to vary, alter, or revoke, and make such new and other bye-laws as they shall think most useful and expedient, so that the same be not repugnant to these presents or to the laws of this our realm: Provided that no bye-law hereafter to be made, or alteration or repeal of any bye-law which shall hereafter have been established by the said council hereby directed to be appointed, shall be considered to have passed and be binding on the said society, until such bye-laws, or such alteration or repeal of any bye-laws, shall, after such notice to the fellows as from time to time may be deemed expedient by the said society, have been confirmed by ballot by the members at large of the said society, ten at least of the fellows of the said society being present; and provided that no such bye-law, or alteration or repeal of any bye-law, shall be deemed or taken to pass in the affirmative, unless it shall appear upon such ballot that not less than two thirds of the fellows present at such meeting shall have voted for the same. And our further will and pleasure is, that it shall be lawful for any three fellows, by writing under their hands, transmitted to the president or such other officer or officers as may by the bye-laws hereafter to be designated for the purpose, to recommend to the council any new bye-laws, or alteration or repeal of any existing bye-laws; and in case the council shall not agree to such new bye-laws, or alteration or repeal of any existing bye-laws, then our will and pleasure is, that such propositions shall, if required by the said three fellows, be submitted to the consideration of the society at large, and determined on by them in the same way as has been directed with regard to new bye-laws, or alterations or repeals of existing bye-laws which have been approved by the council. In witness whereof we have caused these our letters to be made patent. Witness ourself, at our palace at Westminster, this 30th day of September, in the fifth year of our reign.

By writ of privy seal, EDMUNDS.

